# MicroRNA Expression in the Aqueous Humor of Patients with Diabetic Macular Edema

**DOI:** 10.3390/ijms21197328

**Published:** 2020-10-03

**Authors:** Giuseppina Emanuela Grieco, Guido Sebastiani, Chiara Maria Eandi, Giovanni Neri, Laura Nigi, Noemi Brusco, Romina D’Aurizio, Matteo Posarelli, Tommaso Bacci, Elena De Benedetto, Mario Fruschelli, Maurizio Orlandini, Federico Galvagni, Francesco Dotta, Gian Marco Tosi

**Affiliations:** 1 Diabetes Unit, Department of Medicine, Surgery and Neurosciences, University of Siena, Viale Bracci, 16, 53100 Siena, Italy; giusy.grieco.90@gmail.com (G.E.G.); sebastianiguido@gmail.com (G.S.); launigi@gmail.com (L.N.); noemibrusco91@gmail.com (N.B.); 2 Fondazione Umberto Di Mario ONLUS c/o Toscana Life Sciences, 53100 Siena, Italy; 3 Department of Surgical Science, University of Torino, 10124 Torino, Italy; chiara.eandi@unito.it; 4 Department of Ophthalmology, University of Lausanne, Jules-Gonin Eye Hospital, 1004 Lausanne, Switzerland; 5 Ophthalmology Unit of the Department of Medicine, Surgery and Neuroscience, University of Siena, 53100 Siena, Italy; gio.neri2009@libero.it (G.N.); mposarelli@gmail.com (M.P.); osammot.iccab@gmail.com (T.B.); elenadebenedetto1992@gmail.com (E.D.B.); mario.fruschelli@unisi.it (M.F.); 6 Institute of Informatics and Telematics, National Research Council, 56124 Pisa, Italy; romina.daurizio@gmail.com; 7 Department of Biotechnology, Chemistry and Pharmacy, University of Siena, 53100 Siena, Italy; maurizio.orlandini@unisi.it (M.O.); federico.galvagni@unisi.it (F.G.)

**Keywords:** type 2 diabetes mellitus, diabetic retinopathy, Diabetic Macular Edema, microRNA, miRNA, aqueous humor

## Abstract

We identified and compared secreted microRNA (miRNA) expression in aqueous humor (AH) and plasma samples among patients with: type 2 diabetes mellitus (T2D) complicated by non-proliferative diabetic retinopathy (DR) associated with diabetic macular edema (DME) (DME group: 12 patients); T2D patients without DR (D group: 8 patients); and non-diabetic patients (CTR group: 10 patients). Individual patient AH samples from five subjects in each group were profiled on TaqMan Low Density MicroRNA Array Cards. Differentially expressed miRNAs identified from profiling were then validated in single assay for all subjects. The miRNAs validated in AH were then evaluated in single assay in plasma. Gene Ontology (GO) analysis was conducted. From AH profiling, 119 mature miRNAs were detected: 86 in the DME group, 113 in the D group and 107 in the CTR group. miRNA underexpression in the DME group was confirmed in single assay for let-7c-5p, miR-200b-3p, miR-199a-3p and miR-365-3p. Of these four, miR-199a-3p and miR-365-3p were downregulated also in the plasma of the DME group. GO highlighted 54 validated target genes of miR-199a-3p, miR-200b-3p and miR-365-3p potentially implied in DME pathogenesis. Although more studies are needed, miR-200b-3p, let-7c-5p, miR-365-3p and miR-199a-3p represent interesting molecules in the study of DME pathogenesis.

## 1. Introduction

Diabetic retinopathy (DR) represents a highly specific vascular complication of type 1 (T1D) and type 2 (T2D) diabetes and is the leading cause of visual impairment in working-age adults [1]. Chronic hyperglycemia activates different pathways, involving multiple cytokines, eventually leading to the dysfunction of the retinal neuronal-glial-vascular unit [2]. Vascular endothelial growth factor A (VEGFA) is the most studied cytokine implicated in DR pathogenesis. However, growing evidence shows that the processes triggered by chronic hyperglycemia are more complex and may not be driven by VEGFA alone [2,3,4,5,6].

As a matter of fact, not only protein-coding genes but also noncoding RNAs (ncRNAs) are involved in DR molecular signaling interplay, thus rendering understanding of this condition even more complex [7,8].

MicroRNAs (miRNAs) are short (about 22 nucleotides long) and highly conserved sequences of endogenous ncRNAs that represent a powerful class of gene modulators involved in the main biological processes, including cell growth, differentiation and apoptosis. MiRNAs have very long half-lives and have been found to be stable in many biological fluids, including human serum, plasma, urine, saliva, tears, aqueous humor (AH) and vitreous humor [7,9].

With the aim of identifying new biomarkers and possible additional therapeutic targets, numerous miRNAs have been studied in DR, but this has mainly been performed using cell lines and/or animal models, and only rarely on human samples [10]. In fact, human plasma/serum sampling has only been performed in a few case-control studies recruiting either T1D or T2D patients [11,12,13,14,15]. Investigations using human intraocular samples (vitreous and aqueous humor) are even rarer. Gomaa et al. [16] sampled the vitreous, evaluating miR-200b expression in both T1D and T2D patients, while Usui-Ouchi et al. [17], Hirota et al. [18] and Mammadzada et al. [19] evaluated the expression of multiple miRNAs through a microarray or PCR array vitreal profile. Recently, Chen et al. [20] performed next generation sequencing to evaluate multiple miRNA expression in AH of T2D patients. In the above-mentioned vitreal studies and in the aqueous study, patients recruited were affected by proliferative diabetic retinopathy (PDR). However, based on results in human serum [11,13] and in animal models of diabetic retinopathy [2,3,4,5,6,7], different stages of DR might be associated with different expression of both miRNAs and cytokines, leading researchers to propose a “liquid biopsy” immediately prior to the first intraocular injection in order to identify the predominant pathogenic pathway in a patient-specific manner [21]. To our knowledge, only Cho and colleagues [22] evaluated miRNA expression in intraocular samples of patients suffering from non-proliferative diabetic retinopathy (NPDR) complicated by diabetic macular edema (DME). This study was performed by a PCR array composed of 84 miRNAs, chosen for their association with inflammation and angiogenesis, and conducted using cataract patients as control subjects. However, an unbiased miRNAs profiling in a similar population has not been performed yet.

In order to increase the knowledge of this stage of DR, we performed a TaqMan array profiling of 378 miRNAs in the aqueous humor of patients with T2D-associated NPDR complicated by DME; in T2D patients without DR; and in patients without diabetes. In addition, differentially expressed microRNAs of interest were analyzed in plasma samples from the same patients in order to identify miRNAs, whose expression in plasma could mirror that occurring in aqueous humor.

## 2. Results

The baseline characteristics of the patient groups studied are summarized in Table 1. No between-groups differences were present for age at sampling, Body Mass Index (BMI), or comorbidities.

**Figure 1:**
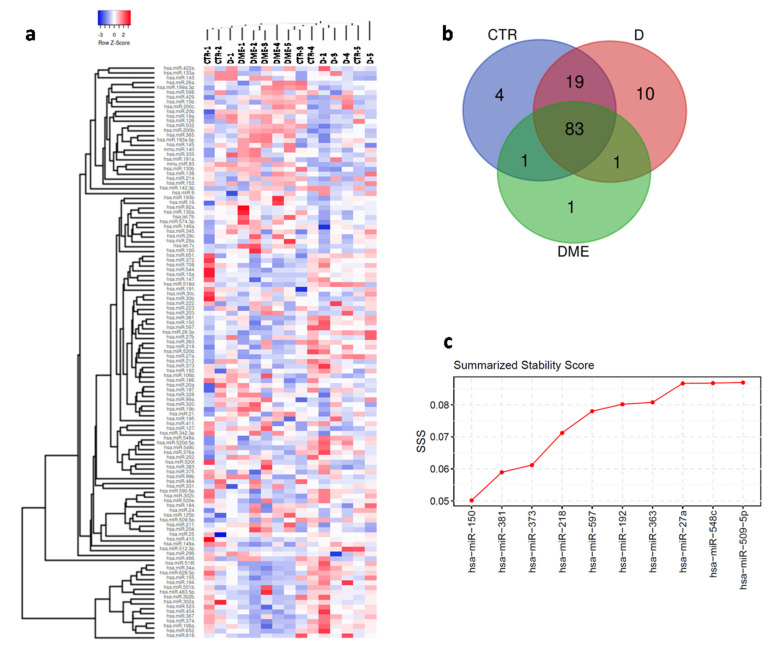
(**a**) Hierarchical Clustering Heatmap analysis of miRNAs detected (*n* = 119) in at least 4/5 sample for each group. A total of 15 subjects were analyzed: *n* = 5 non diabetic controls (CTR), *n* = 5 type 2 diabetic patients (D) and *n* = 5 type 2 diabetic with Diabetic Macular Edema patients (DME). MicroRNAs expression levels are reported as scale colors based on ΔCT expression (Blue, high expression: ΔCT = −3; Red, low expression: ΔCT = 2. (**b**) Venn diagram showing miRNAs distribution among the three groups; in particular, 83 miRNAs were common among the three groups, while 19 miRNAs were exclusively found in CTR and D but not in DME group; moreover, four miRNAs were exclusively detected in CTR groups and 10 miRNAs resulted exclusively expressed in D group, while only 1 miRNA is exclusively detected in DME group. (**c**) Stability ranking of top 10 candidate reference miRNAs based on SSS metric which combined three different assessment algorithms. Most stable miRNAs presented lowest SSS values.

miRNA expression profiling was performed in AH samples using TaqMan miRNA arrays, which allowed the evaluation of 378 miRNA expression levels in *n* = 5 patients per group. Each miRNA analyzed was considered detected when resulting cycle threshold (Ct) was ≤35.0 in at least 4 out of 5 subjects in at least one group. Overall, based on these criteria, a total of 119 miRNAs were reliably detected in AH samples (Figure 1a and Supplementary File S1).

In particular, among the 119 detected miRNAs, 83 were commonly identified in all three groups, while 19 resulted commonly detected between CTR and D groups. Furthermore, 4 miRNAs resulted exclusively expressed in CTR patients, 10 miRNAs were identified only in D group and 1 miRNA was exclusively detected in DME group (Figure 1b).

Considering each group separately, 107/378 miRNAs were detected in the CTR group, 113/378 in the D group and 86/378 miRNAs in the DME group (Supplementary Table S2), highlighting a similar output between CTR and D groups (in terms of miRNAs number), and a significant lower number of miRNAs detected in DME AH samples respect to both CTR and D (Supplementary Table S2 and Supplementary File S1).

In order to compare miRNAs expression levels among the analyzed groups and reduce RT-qPCR data variation, we implemented a normalization step by applying two different strategies: (i) global mean normalization; (ii) best reference miRNAs through combined algorithms normalization. Consequently, the three best-performing miRNAs (miR-150-5p, miR-381-3p and miR-373-3p) (Figure 1c and Supplementary Figure S1a–c) according to SSS metric were chosen to normalize miRNAs expression levels. Therefore, differentially expressed miRNAs were selected from among those identified upon normalization using both strategies.

**Figure 2:**
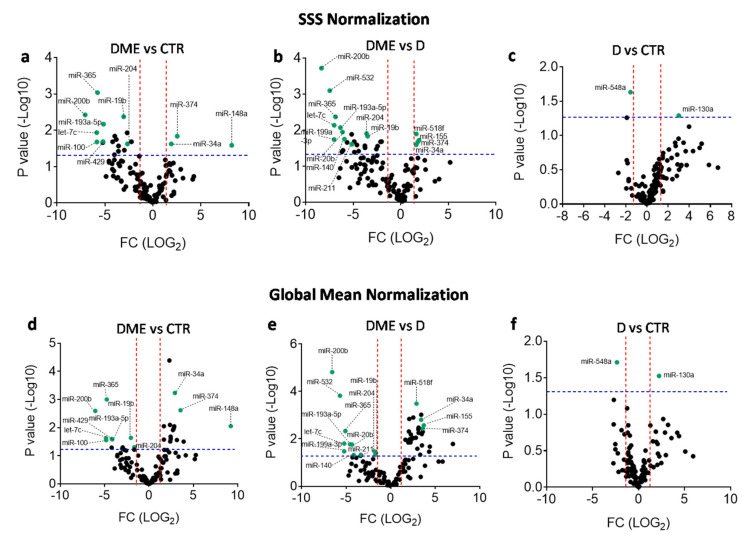
Volcano plots showing differentially expressed miRNAs according to two different normalization strategies. (**a**–**c**) Volcano plots of differentially expressed miRNAs in DME vs CTR (**a**), DME vs. D (**b**), and D vs. CTR patient groups (**c**) computed using SSS normalization strategy. (**d**–**f**) Volcano plots of differentially expressed miRNAs in DME vs. CTR (**d**), DME vs. D (**e**), and D vs. CTR patient groups (**f**) computed using Global Mean normalization strategy. Differentially expressed miRNAs found for both normalizations are labeled and indicated as green dots. Fold change cutoff (red lines) was set at 2.5-fold while *p*-values cutoff (blue line) was set at 0.05 based on Student’s t test on normally distributed ΔCT.

**Figure 3:**
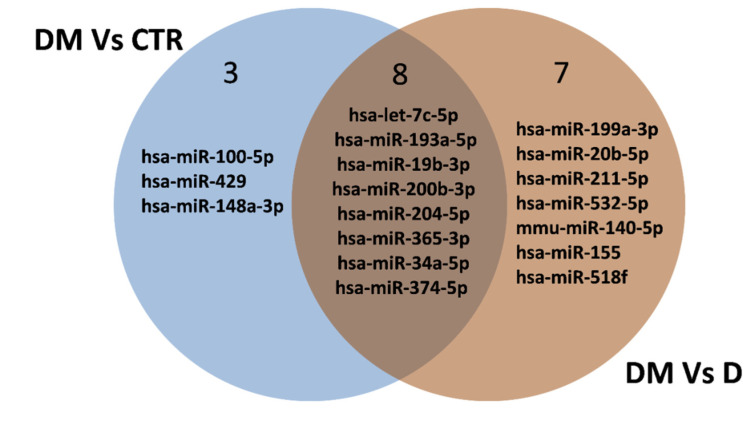
Venn diagram showing the differentially expressed miRNAs among the comparisons of DME vs CTR and of DME vs. D. Eight miRNAs resulted commonly differentially expressed among the comparisons of DME vs. CTR and of DME vs. D (*n* = 6 downregulated miRNAs and *n* = 2 upregulated miRNAs).

According to such criteria, 11 miRNAs were differentially expressed between DME and CTR groups (3 miRNAs upregulated and 8 downregulated in the DME group) (Figure 2a,d and Table 2) and 15 between the DME and D groups (4 miRNAs upregulated and 11 downregulated in the DME group) (Figure 2b,e, and Table 2), while 2 miRNAs were differentially expressed between D and CTR groups (miR-548a-3p downregulated and miR-130a-3p upregulated in D group vs. CTR) (Figure 2c,f). Interestingly, among these miRNAs, 8 resulted commonly differentially expressed among the comparisons DME vs CTR and DME vs D (Table 2 and Figure 3).

### 2.1. miRNAs Downregulation in AH of DME Patients

**Figure 4:**
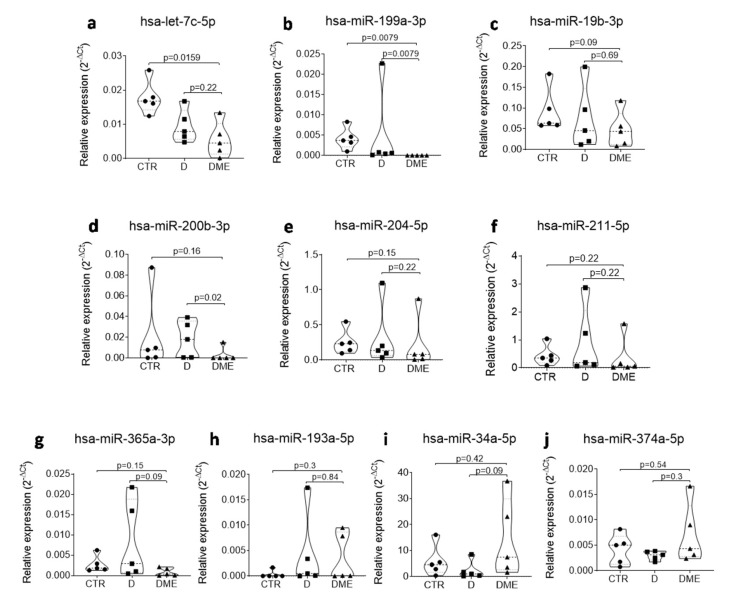
Single assay validation of *n* = 10 microRNAs that resulted deregulated in DME vs CTR and/or vs D in AH of the profiling cohort (5 patients per group) of DME vs CTR and vs D patients. Violin plots show the expression and the relative *p* values of hsa-let-7c-5p (**a**), hsa-miR-199a-3p (**b**), hsa-miR-19b-3p (**c**), hsa-miR-200b-3p (**d**), hsa-miR-204-5p (**e**), hsa-miR-211-5p (**f**), hsa-miR-365-3p (**g**), hsa-miR-193a-5p (**h**), hsa-miR-34a-5p (**i**) and hsa-miR-374a-5p (**j**). miRNA expression values are reported as 2^−ΔCT^ normalized for the expression of hsa-miR-150-5p. Median values for each group are shown as dotted line alongside with quartiles (smaller dotted lines). *p*-values were considered significant with *p* < 0.05 using Mann–Whitney U Test.

**Figure 5:**
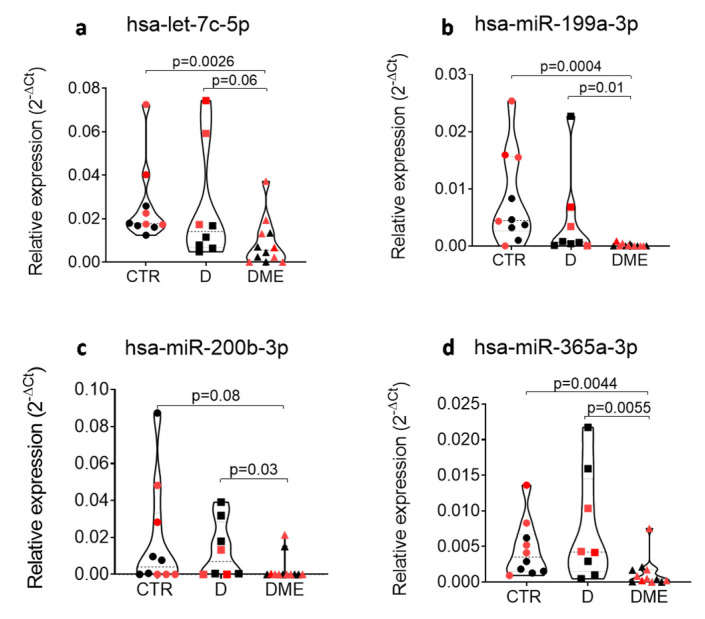
The expression of hsa-let-7c-5p (**a**), hsa-miR-199a-3p (**b**), hsa-miR-200b-3p (**c**) and hsa-miR-365-3p (**d**) is reduced in AH of DME vs CTR and/or vs D patients after testing for all subjects included in the study (*n* = 10 CTR, *n* = 8 D, *n* = 12 DME). Profiling cohort (*n* = 5 CTR, *n* = 5 D, *n* = 5 DME) is indicated with black dots, while the additional validation cohort (*n* = 5 CTR, *n* = 3 D, *n* = 7 DME) is shown as red dots. microRNA expression values are reported as 2^−ΔCT^ normalized for the expression of hsa-miR-150-5p. Median values for each group are shown as dotted line alongside with quartiles (smaller dotted lines). *p*-values were considered significant with *p* < 0.05 using Mann–Whitney U Test.

Among the differentially expressed miRNAs in DME vs. CTR and in DME vs. D, eight were common to both comparisons, while two were specific to the comparison DME vs. D, thus rendering them specifically altered in the AH of DME patients. Therefore, we focused on single assay validation on this set of 10 miRNAs in the same subjects per group as analyzed in the profiling stage (Figure 4). Four miRNAs out of ten were not validated in single assay since they did not confirm the trend observed in the profiling analysis (miR-204-5p, miR-211-5p, miR-193a-5p and miR-19b-3p) (Figure 4), while six out of ten miRNAs were validated in single assay in the five subjects per group (let-7c-5p, miR-200b-3p, miR-199a-3p, miR-365-3p, miR-34a-5p and miRNA-374-5p) and were then tested in all the subjects included in the study (*n* = 10 CTR, *n* = 8 D, *n* = 12 DME). miR-34a-5p and miR-374-5p did not pass the validation in a single assay in all subjects (Supplementary Figure S2), while 4 miRNAs out of 10 were confirmed to be differentially expressed in all subjects and resulted all downregulated in DME group vs CTR or vs. D (let-7c-5p, miR-200b-3p, miR-199a-3p and miR-365-3p) (Figure 5).

### 2.2. miR-199a-3p and miR-365-3p Differential Expression in AH is Mirrored in Plasma Samples

**Figure 6:**
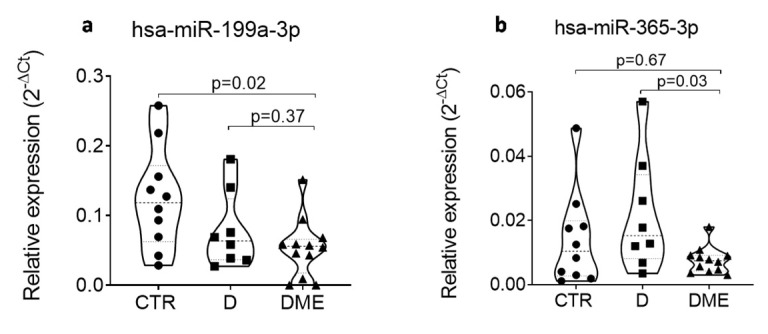
The expression of hsa-miR-199a-3p (**a**) is reduced in plasma of DME (*n* = 12) vs. CTR (*n* = 10) patients; the expression of hsa-miR-365-3p (**b**) is reduced in plasma of DME (*n* = 12) vs. D (*n* = 8) patients. miRNA expression is indicated as 2^−ΔCT^ normalized for the expression of hsa-miR-191-5p and hsa-miR-320a-3p. Median values for each group are shown as dotted line alongside with quartiles (smaller dotted lines). *p*-values were considered significant with *p* < 0.05 using Mann–Whitney U Test.

Since we confirmed the differential expression of these four miRNAs in AH samples of DME patients, we hypothesized a systemic alteration of these miRNAs, thus rendering them easily accessible biomarkers. To investigate this hypothesis, we tested them in plasma samples of the same subjects using single assay RT-qPCR. No differential expression for let-7c-5p and miR-200b-3p was observed (Supplementary Figure S3). However, downregulation of miR-199a-3p and miR-365-3p in the plasma of the DME patients was observed (Figure 6), in line with the same reduction observed in the AH samples. However, no significant correlation resulted between the AH and plasma samples of the same subjects (Supplemental Figure S4).

### 2.3. miR-199a-3p and miR-200b-3p Are Involved in Retina-Vascular and Epithelial Cells Homeostasis

In order to functionally correlate AH miRNAs dysregulation to DME pathological alterations, we performed a Gene Ontology (GO) analysis using DAVID 6.8 algorithm on validated target genes of the 4 downregulated miRNAs in the AH of DME patients identified through miRPath 3.0 algorithm. No validated target gene was identified for let-7c-5p, while *n* = 1614 validated target genes were identified for overall miR-199a-3p, miR-200b-3p and miR-365-3p (Supplementary File S2). GO bioinformatic analysis results on 1613 genes recognized by DAVID 6.8 algorithm (Supplementary File S2) highlighted that 54 validated target genes of miR-199a-3p, miR-200b-3p and miR-365-3p are significantly involved in 8 pathways that could be potentially implied in the pathogenesis of diabetic macular edema (Table 3), including VEGFA signaling pathway (*n* = 19 genes/1613, Fold enrichment = 2.4, Fisher’s exact test corrected *p* value = 0.00021). Interestingly, we also observed that validated target genes of miR-199a-3p, miR-200b-3p and miR-365-3p are involved in establishment of endothelial barrier signaling pathway (*n* = 9 genes/1613, 0.6%, Fold enrichment = 3.0, Fisher’s exact test corrected *p* value = 0.0024), which includesF11 receptor among the validated target genes of miR-199a-3p.

Accordingly, the correlation between VEGFA expression and both miR-199a-3p and miR-200b-3p was checked measuring VEGFA levels in the AH. Due to lack of residual AH, VEGFA concentrations were measured only in *n* = 9 CTRs, *n* = 6 Ds and *n* = 11 DMEs. However, no correlation between VEGF and either miR-199a-3por miR-200b-3p was found (data not shown).

Since F11 receptor seems to be implicated in the maintenance of retinal pigment epithelium barrier, we then checked whether DME patients showing serous retinal detachment (SRD) at OCT examination showed also miR-199a-3p differential expression levels in AH respect to those not showing any SRD. Five out of 12 DME patients presented DME associated with SRD (42%). However, miR-199a-3p expression levels were not different between DME patients showing SRD vs. those without SRD (data not shown).

## 3. Discussion

The peculiarity of the present study starts from the population examined: as a matter of fact, AH miRNAs were evaluated in T2D patients affected by DME. Previous studies evaluating miRNA expression through intraocular sampling are few in number and all but one were conducted on patients affected by PDR, in the context of either type 1 or type 2 diabetes [16,17,18,19,20]. We enrolled only patients showing DME with mild or moderate NPDR in an attempt to minimize the ischemic component and consequently the molecular pathways driven by ischemia. DME associated with PDR would probably have provided new information in any way, but interpretation of the results would have been more difficult due to the ischemic component that characterizes PDR. In fact, based on results in human serum [11,23] and in animal models of diabetic retinopathy [2,3,4,5,6,7], different stages of DR seem to be associated with different expression of both miRNAs and cytokines. Moreover, we enrolled only T2D patients on the basis of the different etiopathological mechanisms that distinguish T2D from T1D [24]. Cho and colleagues [22] were the only ones to have previously studied miRNA expression in DME in the context of NPDR without specifying if patients were affected by T1D or T2D. Additionally, we compared miRNA expression in DME patients not only with that in patients without diabetes but also with that in patients with diabetes but without DR, in order to verify whether or not diabetes per se could be associated with a particular pattern of intraocular miRNA expression and to study its impact on the DME miRNA profile.

In the present series both AH and plasma samples were collected at entry from all the patients enrolled. However, since local biomarkers seem to reflect the pathogenic events that are taking place in the retina more directly compared to circulating biomarkers [21,25], we decided to first profile and validate the differential expression of miRNAs in the AH and then to verify their concomitant differential expression in the plasma.

AH samples have been previously studied to verify different miRNA expression in open-angle glaucoma as compared to the expression in cataract patients [26,27,28,29]. Cataract patients also constituted the control group in the present analysis. We detected 107 mature miRNAs in the present CTR population, 97 of which (91.5%) were shared with the CTR group of Jayaram et al. [26], who also performed their evaluation through microarray analysis.

In patients affected by PDR different miRNA disregulation has been found. Gomaa et al. [16] found miR-200b to be upregulated in both T1D and T2D diabetic patients [16]. Usui-Ouchi et al. [17] identified miR-21 (upregulated in the vitreous of diabetic patients) as potential disease-modifying agent for the development of proliferative disease. Hirota et al. [18] investigated the expression of 168 miRNAs in the vitreous humor of patients with PDR and found that six miRNAs (miR-15a, miR-320a, miR-320b, miR-93, miR-29a and miR-423-5p), related to angiogenesis and fibrosis, are significantly overexpressed in PDR. Mammadzada et al. [19] showed a significant increase in the miRNA-19a, miR-27a, miR-20a and miR-93 expression in PDR patients undergoing PPV as compared to the controls. Recently by next generation sequencing, Chen et al. [20] found seven miRNAs upregulated (miR-150-5p, miR-30c-5p, miR16-2-3p, miR-1827, miR-140-3p and miR-93-5p) and one downregulated (miR-99-5p) in AH of T2D patients with PDR as compared to controls.

In the only study in DME patients, through PCR array of 84 miRNAs, Cho et al. [22] found a significant downregulation of 59 miRNAs in patients as compared to controls. Among the significantly dysregulated miRNAs, five (miR185-5p, hsa-miR-17-5p, hsa-miR-20a-5p, hsa-miR-15b-5p and hsa-miR-15a-5p) were further tested using real-time PCR and all were found to be downregulated.

The four previous studies evaluating miRNA expression in the vitreous of PDR patients relied on patients affected by macular hole as the control group [16,17,18,19]. Chen et al. [20], in the evaluation of miRNAs expression of PDR patients, and Cho et al. [22], in the evaluation of miRNA expression in the AH of DME patients, used cataract patients as the control group. Therefore, this is the first study to evaluate intraocular miRNA expression in diabetic patients without DR and/or DME. From our results, diabetes per se does not seem to be characterized by a different AH miRNA profile compared to that of the cataract-only group, since the mature miRNAs detected after profiling was highly similar between the two groups. On the contrary, the DME group showed miRNA under-expression compared to both the CTR and D groups (86 miRNA were detected in the DME group vs. 113 in the D group and 107 in the CTR group), which was confirmed in a single assay since miR-200b-3p, let-7c-5p, miR-365-3p and miR-199a-3p were found to be significantly downregulated. As such, different intraocular miRNA expression seems to occur in the diabetic patient only after the process of diabetic retinopathy has begun.

In the present study we show that miR-200b, let-7c, miR-365 and miR-199a-3p were all downregulated in DME patients. If we compare our results with those of Cho et al. [22], in both studies a general downregulation of miRNAs in DME patients as compared to controls is found; however, the single miRNAs identified as downregulated are different. The discrepancies in the differentially expressed miRNAs identified between the two studies might be explained by the different methods of analysis used by Cho et al. Indeed, the authors analyzed the expression of 84 miRNAs among those most studied and already reported to be involved in angiogenesis and inflammation. Moreover, the method of normalization for the evaluation if miRNAs expression is not completely appropriate given the use of the small nucleolar RNU6-6p (which is not a miRNA).

miR-200b, let-7c, miR-365 and miR-199a-3p have already been found to be implicated in DR—miR-200b being the only of the four to have been analyzed on human intraocular samples. However, their protective or aggravating role reported can hardly be compared with their downregulation shown in the present series, since the animal models, being devoid of the macula region, cannot fully mimic the course of DR and the eye’s molecular behavior in humans, and since discrepancies in their expression have been reported even when different animal models were used [7,30,31,32]. The prototype of these previous conflicting results is miR-200b, which has been found to be downregulated in endothelial cells under hyperglycemia in the retinas of STZ-induced diabetic rats and in the sera of DR patients, whereas it is upregulated in the retinas of a genetic model of type 1 diabetes (Akita mice) and the vitreous of PDR patients [7]. In the present seriesmiR-200b-3p is downregulated and not upregulated, as shown by Gomaa et al. [16] in their PDR patients; however, again, we included only patients affected by NPDR. In accordance with our results, both let-7c and mir-365 were found to be implicated in the early stages of the DR animal model but, in contrast to our results, rather than a protective role, they were shown to have an aggravating role: increased vessel tortuosity and decreased pericyte coverage for let-7c and increased oxidative stress for miR-365 [30,31,32]. In relation to the previously reported expression of miR-365, the discrepancy with the present results is even greater, since the downregulation found in the AH of our patients was also confirmed in their plasma. The only miRNA whose previously reported role is in line with that reported herein is miR-199a-3p. Although implicated in a variety of carcinomas as either a repressor or promoter, miR-199a-3p has been found to be downregulated in the animal model of DR, thus confirming the protective role in both the AH and plasma reported here [7,33].

GO bioinformatic analysis highlighted that 54 validated target genes of miR-199a-3p, miR-200b-3p and miR-365-3p are significantly involved in eight pathways that could be potentially implied in the pathogenesis of diabetic macular edema. The only soluble mediators associated with these 8 pathways (and therefore measurable in AH) are VEGFA, TGFβ1 and Gremlin-1, an inhibitor of TGFβ pathway. Since we recently showed that TGFβ1 is poorly expressed in the AH [34,35], we decided to measure VEGFA concentration. Additionally, GO analysis identified a direct validated regulation of miR-199a-3p on F11 receptor, which has been shown to be involved in the maintenance of the retinal pigment epithelium barrier [36]. However, no correlation between VEGFA and either miR-199a-3p or miR-200b-3pAH levels was found. This is not surprising, since anti-VEGFA agents show inefficacy in around half of DME patients notwithstanding intensive injection regimens and, as reported by Aiello et al. [37], 36% of diabetic patients with PDR had undetectable levels of VEGFA in the vitreous fluid [3,4,21]. F11R/JAM-A is a tight junction protein that is important for platelet and leukocyte interactions with the epithelium and endothelium and has been found to be expressed by the retinal pigment epithelium [36]. Accordingly, it might be implicated in the regulation of this important physiological barrier and in the occurrence of macular edema, and in particular with the DME subtype associated with SRD. Although the pathogenetic theories at the basis of SRD occurrence are different, the role of RPE in retinal fluid homeostasis seems to be clinically confirmed by the results of the present series, since the DME patients showed a SRD incidence of 42%, which is in the high range compared to the 15%–45% reported in the literature [38,39,40]. However, we failed to demonstrate a significant differential expression of each of the downregulated miRNAs, including miR-199a-3p, between patients with and without SRD.

The site of intraocular sampling may represent a potential limitation of the present study. Sampling of vitreous humor could be more informative for patients affected by DR and DME; however, due to ethical reasons, AH is nowadays considered the most appropriate biological sample for the investigation of intraocular biomarkers in DME. The formation of cataract might have had an effect on our results in CTR and D patients; however, no difference in age was present between the three groups.

In conclusion, different intraocular miRNA expression seems to occur in diabetic patients only after the process of diabetic retinopathy has begun. In patients affected by NPDR complicated by DME, miR-200b-3p, let-7c-5p, miR-365-3p and miR-199a-3p were downregulated in the AH, and miR-365-3p and miR-199a-3p were also downregulated in the plasma. Additional studies are needed to understand the complicated molecular interplay occurring in DME and to verify the role of miR-199a-3p in relation to the retinal pigment epithelial barrier.

## 4. Materials and Methods

### 4.1. Subjects

The present study was performed on three groups of patients: *n* = 12 T2D patients with DR complicated by DME (DME group), *n* = 8 T2D patients without any sign of DR (D group), and *n* = 10 non-diabetic patients (CTR group). All eyes were examined and treated between September 2016 and March 2018 at the Ophthalmology Unit of the Department of Medicine, Surgery and Neuroscience, Siena University Hospital, Siena, Italy, following approval by the Institutional review board C.E.A.V.S.E. (Ethical Committee for clinical spermientation, South-Eastern Tuscany) (identification code MIRNADR16, approval date 16 May 2016). The study complied with the Declaration of Helsinki and patients were treated after being informed of the nature, purpose, implications and risks of the treatment and after having signed a consent form.

Patient demographics were recorded, including length of diabetic history, glycosylated hemoglobin (HbA1c), body mass index (BMI) and systemic medications. Ophthalmological examination included best-corrected visual acuity (BCVA) by Snellen chart recording (and conversion to logMAR for statistical analysis) and intraocular pressure measurement, anterior segment slit lamp examination, dilated fundus examination with slit-lamp biomicroscopy and indirect ophthalmoscopy, as well as spectral-domain OCT central subfield thickness (CST) (Cirrus HD-OCT; Carl Zeiss Meditec AG, Jena, Germany). The baseline severity of DR and the diagnosis of center-involving macular edema among the eyes enrolled were evaluated following clinical and OCT examinations according to the International Council of Ophthalmology recommendations [1].

The inclusion criteria were: (i) for the DME group—being affected by T2D complicated by mild to moderate NPDR associated with treatment-naive center-involving DME with a CST of >310 μm; (ii) for the D group—being affected by T2D without signs of DR and/or DME; (iii) for the CTR group—not being affected by T2D. All patients included were phakic with a mild to moderate cataract. For the DME group whenever DME was bilaterally present, the eye with a higher CST determined by SD-OCT was selected as the study eye.

The exclusion criteria were: race other than Caucasian; types of diabetes other than T2D and presence or history of underlying connective tissue, inflammatory or malignant disease; presence of hepatic insufficiency, renal impairment and infectious diseases; ischemic heart attack in the previous 12 months; diabetic neuropathy; being affected by severe non-proliferative or proliferative DR or by any other ocular disease except mild to moderate cataract for the DME group; being affected by any other ocular disease except mild to moderate cataract for the D group and CTR groups; prior intraocular surgery of any kind, including cataract surgery; and history of any intravitreal injection, ocular trauma or laser treatment.

### 4.2. AH and Plasma Sample Collection and Processing

Approximately 50 to 100 μL of AH were collected from each patient through a clear corneal paracentesis using a 30-gauge needle just before the intravitreal injection (DME group) or prior to placement of the initial cataract incision (D and CTR group). AH samples were collected in 0.5 mL nuclease-free microtubes, stored on-ice and further processed within 2 h from collection. Sample collection was atraumatic in all cases, thereby eliminating the risk of contamination with blood or cellular debris. Eventual cells/cellular debris contamination was prevented by centrifuging AH samples at 1200× *g* for 20 min at 10 °C. Supernatant was then transferred to another tube and stored at −80 °C until ready for RNA extraction.

A SOP was followed to collect plasma samples as previously described [41]. Blood was collected in BD Vacutainer K_2_-EDTA tubes (BD Biosciences, San Jose, CA, USA), inverted 5 times and stored upright at room temperature until ready for processing. Blood samples were processed within 2 h from blood draw by centrifugation at 1800× *g* for 10 min at room temperature; collected plasma was further centrifuged at 1200× *g* for 20 min at 10 °C in order to remove contaminant cells and cellular debris. Finally, plasma samples were aliquoted and subsequently stored at ‒80 °C. Hemolyzed plasma samples were excluded from the study.

### 4.3. RNA Extraction

Total RNA, including small RNAs <200 nt, was extracted from 50 μL of AH or 200 μL of plasma samples using MicroRNA miRNeasy Mini extraction kit (Qiagen, Hilden, Germany) with some modifications. AH and plasma samples were thawed on ice and centrifuged at 3000× *g* for 5 min at 4 °C to completely remove contaminant cellular debris. A total of 50 µL of AH and 200 µL of plasma from each patient were diluted in up to 400 µL in nuclease-free water to avoid protein aggregates. Samples were then lysed by adding 3 volumes of Trizol LS (Life Technologies, Carlsbad, CA, USA) and finally eluted in 30 µL of nuclease-free water.

### 4.4. TaqMan Array miRNA Expression Profiling

TaqMan MicroRNA Human Array Panel A platform (Life Technologies, Carlsbad, CA, USA) was adopted to profile the expression of 378 miRNAs in total RNA extracted from AH samples. RNA was reverse-transcribed using Megaplex RT primers Pool-A (Life Technologies, Carlsbad, CA, USA). Briefly, 3 μL of extracted RNA from 50 μL of each AH sample were added to 0.8 μL of 10× Megaplex RT Primers, 0.2 μL of 100 mMdNTPs, 1.5 μL of 50 U/μL Multiscribe RT, 0.8 μL of 10× RT Buffer, 0.9 μL of 25mM MgCl2, 0.1 μL of 20 U/μL RNAse inhibitor and 0.2 μL of nuclease-free H_2_O. The product of this reaction was incubated for 40 cycles at 16 °C for 2 min, 42 °C for 1 min and 50 °C for 1 s, and then at 85 °C for 5 min. Afterwards, the synthesized cDNA was pre-amplified using Megaplex Preamp primers Pool-A. 2.5 μL of cDNA from each sample were added to 12.5 μL of 2× TaqMan Preamp Master Mix, 2.5 μL of 10× Preamp Primers A V.2.1 and 7.5 μL of H_2_O. The product of this reaction was incubated at 95 °C for 10 min, at 55 °C for 2 min and at 72 °C for 2 min, then for 12 cycles at 95 °C for 15 s and 60 °C for 4 min and, finally, at 99 °C for 10 min. Finally, the preamplified cDNA was diluted 1:4 in 0.1× Tris-EDTA pH8.0 to obtain a final volume of 100 μL.

The reaction mix for each microfluidic card was prepared by adding 360 μL of H_2_O and 450 μL of TaqMan Universal PCR Master Mix 2× to 90 μL of diluted and pre-amplified cDNA. The product of this reaction was incubated at 95 °C for 10 min, followed by 40 cycles of 95 °C for 15 s and 60 °C for 1 min. The Real-Time PCR instrument ViiA7 (Life Technologies, Carlsbad, CA, USA) was used to perform the reactions.

Resulting data were analyzed and exported using Expression Suite 1.2.1 software (Life Technologies, Carlsbad, CA, USA). Comparative analyses among DME, D and CTR groups was performed by using 2^−ΔΔCt^ method following normalization with both Global Mean Normalization (GMN) method and most stable housekeeping miRNAs in AH.

The expression of 10 differentially expressed miRNAs obtained from the profiling in the comparison between the DME and D groups and the DME and CTR groups was validated on the same 5 subjects per group used for the profiling through a single assay RT-qPCR using TaqMan miRNA chemistry. Among the 10 miRNAs chosen, only the miRNAs which confirmed to be differentially expressed in a single assay were further analyzed in single assays in the AH of the whole population. Only the miRNAs validated in the AH of all subjects were then analyzed in a single assay in plasma from the same subjects. Differential miRNA expression was calculated by relative quantification using the comparative Ct method, with normalization performed using miR-150-5p, miR-373-3p and miR-381-3p. The results were expressed as the fold change in DME AH group compared with AH from CTR and D groups, representing the ratio of the mean normalized expression values of both groups.

### 4.5. Selection of Housekeeping miRNAs for AH Samples

To establish candidate endogenous miRNAs to be exploited as reference normalizers we adopted a data-driven approach based on a combination of three different algorithms as described by Marabita et al. [42]. Each of the three algorithms generated a stability score such that a smaller score corresponds to higher expression stability that we combined into the summarized stability score (SSS) to select top candidate miRNAs in all the methods. We considered only miRNAs with complete observations since we wanted to select miRNAs serving as endogenous controls which were abundantly and stably detected in all samples. In more details, to measure the stability of miRNA expression among DME, D and CTR groups we used geNorm [43] and Normfindern [44] which were implemented into the R/Bioconductor package NormqPCR [45] and a CV-based score. For each miRNA, geNorm calculates the pairwise variation (V) with all other miRNAs across the samples and defines a stability score (*M*) as the average V of a particular miRNA with all other control miRNAs. Genes with the lowest M values have the most stable expression. Instead, Normfinder is a model-based approach which estimates the intergroup and intragroup variations of a miRNA, and then combines them into a stability value (*rho*). Then, for each miRNA we calculated the coefficient of variation (CV) as its standard deviation across samples divided by the mean and scaled it by the sum of all CVs for each sample (*CV score*). Lastly, the SSS was calculated as the three-dimension Euclidean distance from the origin, i.e.,

$$SSS=\sqrt{meanM^{2}+rho^{2}+CVscore^{2}}$$

In so doing, SSS combined the results from all the methods and allowed to reveal the most stable housekeeping miRNAs (Figure 1c). The stability scores generated by each algorithm for the top 10 candidate miRNAs are shown in Supplementary Figure S1 while the scores full list is reported in Supplementary File S1.

### 4.6. miRNAs Single Assay RT-qPCR

RNA was reverse-transcribed employing Custom RT primers pool and preamplified using Custom Preamp primers pool. Briefly, 5 µL each RT or TM primer (Supplementary Table S1) was diluted in a total volume of 500 µL of Tris-EDTA 1× and used for RT or preamplification reaction. Then, 3 µL of extracted RNA were added to 6 µL of custom primers pool, 0.30 µL 100 mMdNTPs, 3 µL of 50 U/µL Multiscribe RT, 1.50 µL 10× RT Buffer, 0.19 µL 20 U/µL RNase inhibitor and 1.01 µL H_2_O. The reaction product was incubated at 16 °C for 30 min, 42 °C for 30 min and then at 85 °C for 5 min. Afterwards, the synthesized cDNA was preamplified using Custom Preamp primer pool: 2.5 µL of cDNA from each sample were added to 12.5µL 2× TaqMan Preamp Master Mix, 3.75 µL 10× Custom Preamp primers and 6.75µL nuclease-free H_2_O. The reaction was incubated at 95 °C for 10 min, at 55 °C for 2 min and at 72 °C for 2 min, then for 12 cycles at 95 °C for 15 s and 60 °C for 4 min and, finally, at 99 °C for 10 min. In each well, 5µL of preamplified cDNA (diluted 1:8 in Tris-EDTA 0.1×) were added to 15 µL reaction mix composed of 10 µL TaqMan Universal Master Mix, 1 µL of TaqMan miRNA expression assay and 4 µL of nuclease-free H_2_O. The reaction was incubated at 95 °C for 10 min, followed by 40 cycles at 95 °C for 15 s and at 60 °C for 1minute. ViiA7 Real-Time PCR instrument was used to analyze the reactions while Expression Suite 2.1 software was used to evaluate the amplification plot efficiencies and to analyze expression data. Analysis was performed using 2^−∆Ct^ method upon normalization of raw Ct using expression level of miR-150-5p (for AH samples) which was the most stably expressed among those identified in the profiling stage (Supplementary Figure S1). miRNA miR-320a-3p and miR-191-5p expression levels were adopted to perform normalization in plasma samples as previously shown [46].

### 4.7. Bioinformatic Analysis

miRPath 3.0 algorithm was used to identify validated target genes of the validated differentially expressed miRNAs and Gene Ontology (GO) analysis was performed using DAVID 6.8 algorithm to identify biological processes and specific pathways putatively implicated in the pathogenesis of DME in which the validated target genes of the differentially expressed miRNAs are involved.

### 4.8. Proquantum High-Sensitivity Immunoassay

AH VEGFA concentration was evaluated in *n* = 9 CTR subjects, *n* = 6 D and *n* = 11 DME patients through specific Proquantum VEGFA immunoassay following the manufacturer’s instructions. Briefly, 5 µL of each AH sample diluted 1:3 in assay dilution buffer were added to 5 µL of antibody-conjugate mix (calculated for 26 samples: 12 of antibody-conjugate A, 12 µL of antibody-conjugate B and 696 µL of antibody-conjugate dilution buffer) and incubated 1 h RT. Then, 40 µL of qPCR reaction mix were added to each well and RT-qPCR was performed at the following temperatures: 25 °C for 20 min, 95 °C for 2 min, 40 cycles (95 °C 1 s, 60 °C 20 s). The Real-Time PCR instrument ViiA7 was used to perform the reactions. The subsequent analysis was performed through Proquantum online app (apps.thermofisher.com/apps/proquantum).

### 4.9. Statistical Analysis

Data are reported as means or median ± SD; sample value distribution analysis was performed for all parameters using the D’Agostino–Pearson normality test. The non-parametric Mann–Whitney U Test or Student’s *t*-test was adopted for *p* value evaluation according to the distribution of values; a *p* value ≤ 0.05 was considered as statistically significant. MicroRNA expression levels were reported as normalized 2^−∆Ct^ values compared among the selected groups using the Mann–Whitney U test (*p* value ≤ 0.05).

## Figures and Tables

**Table 1:** Clinical characteristics of patients.

Characteristics	CTR	D	DME
Demographic, *n* =	10	8	12
Age, mean (SD; range), years	71 (4.8; 65–78)	72 (5,1; 65–78)	69 (4.9; 57–79)
**Sex, No. (%)**			
Male	7 (70%)	6 (75%)	8 (67%)
Female	3 (30%)	2 (33%)	4 (33%)
BMI, mean (SD; range)	25 (3.2; 22–33)	28 (2.6, 26–34)	27 (1.8; 23–30)
**Comorbidities, No. (%)**			
No comorbidities	2 (20%)	-	-
HTN	4 (40%)	8 (100%)	12 (100%)
BPH	2 (20%)	3 (37%)	-
MI	1 (10%)	-	2 (17%)
DLP	2 (20%)	2 (25%)	3 (25%)
Others	2 (20%)	2 (25%)	2 (17%)
**Treatment, No. (%)**			
Metformin	-	5 (62%)	5 (42%)
Insulin	-	2 (25%)	7 (58%)
Other diabetic drugs	-	3 (37%)	3 (25%)
HTN drugs	5 (50%)	7 (87%)	11 (92%)
Antiplatelet drugs	1 (10%)	3 (37%)	3 (25%)
BPH drugs	2 (20%)	2 (25%)	-
DLP drugs	2 (20%)	2 (25%)	3 (25%)
Others	2 (20%)	3 (37%)	3 (25%)
**Laterality, No. (%)**			
Right	4 (40%)	4 (50%)	5 (42%)
Left	6 (60%)	4 (50%)	7 (58%)
**Best-corrected Visual Acuity**			
Snellen, mean (range)	20/40 (20/25–20/50)	20/40 (20/32–20/63)	20/40 (20/25–20/100)
LogMAR, mean (SD; range)	0.3 (0.1; 0.1–0.4)	0.3 (0.1; 0.2–0.5)	0.3 (0.2; 0.1–0.7)
**OCT Features**			
Central subfield thickness, mean (SD; range), µm	272.4 (12.3; 251–289)	275 (8.2; 263–289)	447.7 (86.6; 319–604)
**Grading of Diabetic Retinopathy, No. (%)**			
Mild	Not applicable	absent	6
Moderate	Not applicable	absent	6
Severe	Not applicable	absent	absent
Duration of diabetes, mean (SD; range), years	Not applicable	7 (1.5; 5–10)	6 (2.7; 1–11)
HbA1c, mean (SD; range)	Not applicable	7 (0.3; 6.9–7.8)	8.0 (1.23; 6.6–10.2)

CTR: patients without diabetes; D: patients with type 2 diabetes without diabetic macular edema; DME: patients with type 2 diabetes with diabetic macular edema; HTN: hypertension; BPH: Benign prostatic hyperplasia; MI: myocardial infarction; DLP: Dyslipidemia.

**Table 2:** List of differentially expressed miRNAs following profiling analyses in AH of DME vs CTR group (*n* = 11; *n* = 8 downregulated miRNAs and *n* = 3 upregulated miRNAs) and in AH of DME vs. D group (*n* = 15 miRNAs; *n* = 11 downregulated miRNAs and *n* = 4 upregulated miRNAs).

Expressed miRNAs	DME Vs. CTR	DME Vs. D
**Downregulated miRNAs**	hsa-let-7c-5p	hsa-let-7c-5p
	hsa-miR-193a-5p	hsa-miR-193a-5p
	hsa-miR-19b-3p	hsa-miR-19b-3p
	hsa-miR-200b-3p	hsa-miR-200b-3p
	hsa-miR-204-5p	hsa-miR-204-5p
	hsa-miR-365-3p	hsa-miR-365-3p
	hsa-miR-100-5p	hsa-miR-199a-3p
	hsa-miR-429	hsa-miR-20b-5p
		hsa-miR-211-5p
		hsa-miR-532-5p
		mmu-miR-140-5p
**Upregulated miRNAs**	hsa-miR-34a-5p	hsa-miR-34a-5p
	hsa-miR-374-5p	hsa-miR-374-5p

**Table 3:** List and details of most important signaling pathways in which validated target genes of miR-200b-3p, miR-365-3p and miR-199a-3p are involved. The first column indicates the biological process in which a specific number (second column) of validated target genes of miR-200b-3p, miR-365-3p and miR-199a-3p are involved. The following columns list the respective *p* values (third column), fold enrichment (fourth column) and Fisher’s exact test corrected *p* values (fifth column). DLL1: delta like canonical Notch ligand 1; NOTCH1: Notch 1; RBPJ: recombination signal binding protein for immunoglobulin kappa J region; MET: proto-oncogene, receptor tyrosine kinase; FGFR1: fibroblast growth factor receptor 1; KDR: kinase insert domain receptor; THBS1: thrombospondin 1; VEGFA: vascular endothelial growth factor A; AKT1: AKT serine/threonine kinase 1; AMOTL1: angiomotin like 1; NFE2L2: nuclear factor, erythroid 2 like 2; PLCG1: phospholipase C gamma 1; PTGS2: prostaglandin-endoperoxide synthase 2; TGFB1: transforming growth factor beta 1; EPHA2: EPH receptor A2; KLF4: Kruppel like factor 4; EFNA1: ephrin A1; PDCD10: programmed cell death 10; RHOA: ras homolog family member A; F11R: F11 receptor; RAP1B: RAP1B, member of RAS oncogene family; RAP2B: RAP2B, member of RAS oncogene family; RAP2C: RAP2C, member of RAS oncogene family; RAPGEF2: Rap guanine nucleotide exchange factor 2; RAPGEF6: Rap guanine nucleotide exchange factor 6; AFDN: afadin, adherens junction formation factor; MSN: moesin; RDX: radixin; AXL receptor tyrosine kinase(AXL); CRK:CRK proto-oncogene, adaptor protein; NCKAP1: NCK associated protein 1; ACTG1: actin gamma 1; CDC42: cell division cycle 42; ELMO1: engulfment and cell motility 1; ELMO2: engulfment and cell motility 2; FLT1: fms related tyrosine kinase 1; ITGAV: integrin subunit alpha V; NEDD4 neural precursor cell expressed, developmentally down-regulated 4, E3 ubiquitin protein ligase; PAK 2: p21 (RAC1) activated kinase 2; PIK3CB: phosphatidylinositol-4,5-bisphosphate 3-kinase catalytic subunit beta; PIK3R1: phosphoinositide-3-kinase regulatory subunit 1; PRKCB: protein kinase C beta: PTK2: protein tyrosine kinase 2; RAC1: ras-related C3 botulinum toxin substrate 1 (rho family, small GTP binding protein Rac1); AKT serine/threonine kinase 1(AKT1); GREM1: gremlin 1, DAN family BMP antagonist(GREM1); ADAM9: ADAM metallopeptidase domain 9; ETS1: ETS proto-oncogene 1, transcription factor; JUN: Jun proto-oncogene, AP-1 transcription factor subunit; SOX9: SRY-box 9; WNT5A: Wnt family member 5; CAPN7: calpain 7: CLASP1: cytoplasmic linker associated protein 1(CLASP1; DOCK5: dedicator of cytokinesis 5; ZNF580: zinc finger protein 580.

Signaling Pathway	Genes Count	Genes %	Genes ID	*p*-Value	Fold Enrichment	Fisher Exact Test
arterial endothelial cell differentiation	3	0.2	DDL1, NOTCH1, RBPJ	8.90 × 10^−2^	5.8	1.10 × 10^−2^
regulation of endothelial cell chemotaxis	6	0.4	MET, FGFR1, KDR, NOTCH1, THBS1, VEGFA	1.20 × 10^−2^	4.1	2.20 × 10^−3^
positive regulation of blood vessel endothelial cell migration	8	0.5	AKT1, AMOTL1, NFE2L2, PLCG1, PTGS2,THBS1, TGFB1, VEGFA	4.30 × 10^−3^	3.7	8.80 × 10^−4^
regulation of blood vessels endothelial cell migration	14	0.9	AKT1, EPHA2, KLF4, AMOTL1, EFNA1, NOTCH1, NFE2L2, PLCG1, PDCD10, PTGS2, RHOA, THBS1, TGFB1, VEGFA	3.70 × 10^−4^	3.1	9.50 × 10^−5^
establishment of endothelial barrier	9	0.6	F11R, RAP1B, RAP2B, RAP2C, RAPGEF2, RAPGEF6, AFDN, MSN, RDX	8.70 × 10^−3^	3	2.40 × 10^−3^
vascular endothelial growth factor receptor signaling pathway	19	1.2	AXL, CRK, NCKAP1, ACTG1, CDC42, ELMO1, ELMO2, FLT1, ITGAV, KDR, NEDD4, PAK2, PIK3CB, PIK3R1, PRKCB, PTK2, RHOA, RAC1, VEGFA	6.10 × 10^−4^	2.4	2.10 × 10^−4^
blood vessel endothelial cell migration	14	0.9	AKT1, KLF4, AMOTL1, GREM1, KDR, NOTCH1, NFE2L2, PLCG1, PDCD10, PTGS2, RHOA, THBS1, TGFB1, VEGFA	5.70 × 10^−3^	2.3	2.10 × 10^−3^
positive regulation of epithelial cell migration	20	1.2	ADAM9, AKT1, ETS1, JUN, MET, SOX9, WNT5A, AMOTL1, CAPN7 CLASP1, DOCK5, FGFR1, KDR, NFE2L2, PLCG1 PTGS2, THBS1, TGFB1, VEGFA, ZNF580	1.60 × 10^−3^	2.2	6.10 × 10^−4^

## Supplementary Materials

Supplementary materials can be found at https://www.mdpi.com/1422-0067/21/19/7328/s1.

## Abbreviations

miRNA	MicroRNA
AH	Aqueous humor
T2D	Type 2 diabetes mellitus
DR	Diabetic retinopathy
DME	Diabetic macular edema
CTR	Control
GO	Gene ontology
T1D	Type 1 diabetes mellitus
VEGFA	Vascular endothelial growth factor A
PDR	Proliferative diabetic retinopathy
NPDR	Non-proliferative diabetic retinopathy
HbA1c	Glycosylated hemoglobin
BMI	Body mass index
BCVA	Best-corrected visual acuity
LogMAR	Logarithm of the minimum angle of resolution
OCT	Optical coherence tomography
CST	Central subfield thickness
SOP	Standard operating procedure
Ct	Cycle threshold
SRD	Serous retinal detachment
TGFβ1	Transforming growth factor β1
