# Supplementary material for: MicroRNA Expression in the Aqueous Humor of Patients with Diabetic Macular Edema
Source: Int J Mol Sci. 2020 Oct 3;21(19):7328. doi: 10.3390/ijms21197328 (PMC7582592; doi:10.3390/ijms21197328)
Supplement: Supplementary file 1 [file ijms-21-07328-s001.zip › ijms-942167-supplementary da inviare/Supplementary Table S1.docx]

| **miRNA** | **Firm** | **Catalog number** | **ID code** | **Used for** |
| --- | --- | --- | --- | --- |
| hsa-miR-199a-3p | Thermo Fisher Scientific | 4427975 | 002304 | Differential expression analysis |
| hsa-miR-211-5p | Thermo Fisher Scientific | 4427975 | 000514 | Differential expression analysis |
| hsa-let-7c-5p | Thermo Fisher Scientific | 4427975 | 000379 | Differential expression analysis |
| hsa-miR-193a-5p | Thermo Fisher Scientific | 4427975 | 002281 | Differential expression analysis |
| hsa-miR-19b-3p | Thermo Fisher Scientific | 4427975 | 000396 | Differential expression analysis |
| hsa-miR-200b-3p | Thermo Fisher Scientific | 4427975 | 002251 | Differential expression analysis |
| hsa-miR-204-5p | Thermo Fisher Scientific | 4427975 | 000508 | Differential expression analysis |
| hsa-miR-365-3p | Thermo Fisher Scientific | 4427975 | 001020 | Differential expression analysis |
| hsa-miR-34a-5p | Thermo Fisher Scientific | 4427975 | 000426 | Differential expression analysis |
| hsa-miR-374-5p | Thermo Fisher Scientific | 4427975 | 000563 | Differential expression analysis |
| hsa-miR-381-3p | Thermo Fisher Scientific | 4427975 | 000571 | Candidate AH normalizer |
| hsa-miR-373-3p | Thermo Fisher Scientific | 4427975 | 000561 | Candidate AH normalizer |
| hsa-miR-150-5p | Thermo Fisher Scientific | 4427975 | 000473 | AH normalizer |
| hsa-miR-320a-3p | Thermo Fisher Scientific | 4427975 | 002277 | Plasma normalizer |
| hsa-miR-191-5p | Thermo Fisher Scientific | 4427975 | 002299 | Plasma normalizer |

**Supplementary Table S1.** List and technical specifications about RT and TM primers used to perform miRNAs single assay RT-q PCR.
