# Supplementary material for: MicroRNA Expression in the Aqueous Humor of Patients with Diabetic Macular Edema
Source: Int J Mol Sci. 2020 Oct 3;21(19):7328. doi: 10.3390/ijms21197328 (PMC7582592; doi:10.3390/ijms21197328)
Supplement: Supplementary file 1 [file ijms-21-07328-s001.zip › ijms-942167-supplementary da inviare/Supplementary Table S2.docx]

**Supplementary Table S2.** List of microRNAs detected in at least 4 of 5 patients of each group. In the first column is shown the list of 107/378 miRNAs detected in CTR group; in the second column are reported the 113/378 miRNAs identified in D group; in the third column is indicated the list of 86/378 miRNAs detected in DME group.
