# Supplementary figures and images for: MicroRNA Expression in the Aqueous Humor of Patients with Diabetic Macular Edema
Source: Int J Mol Sci. 2020 Oct 3;21(19):7328. doi: 10.3390/ijms21197328 (PMC7582592; doi:10.3390/ijms21197328)

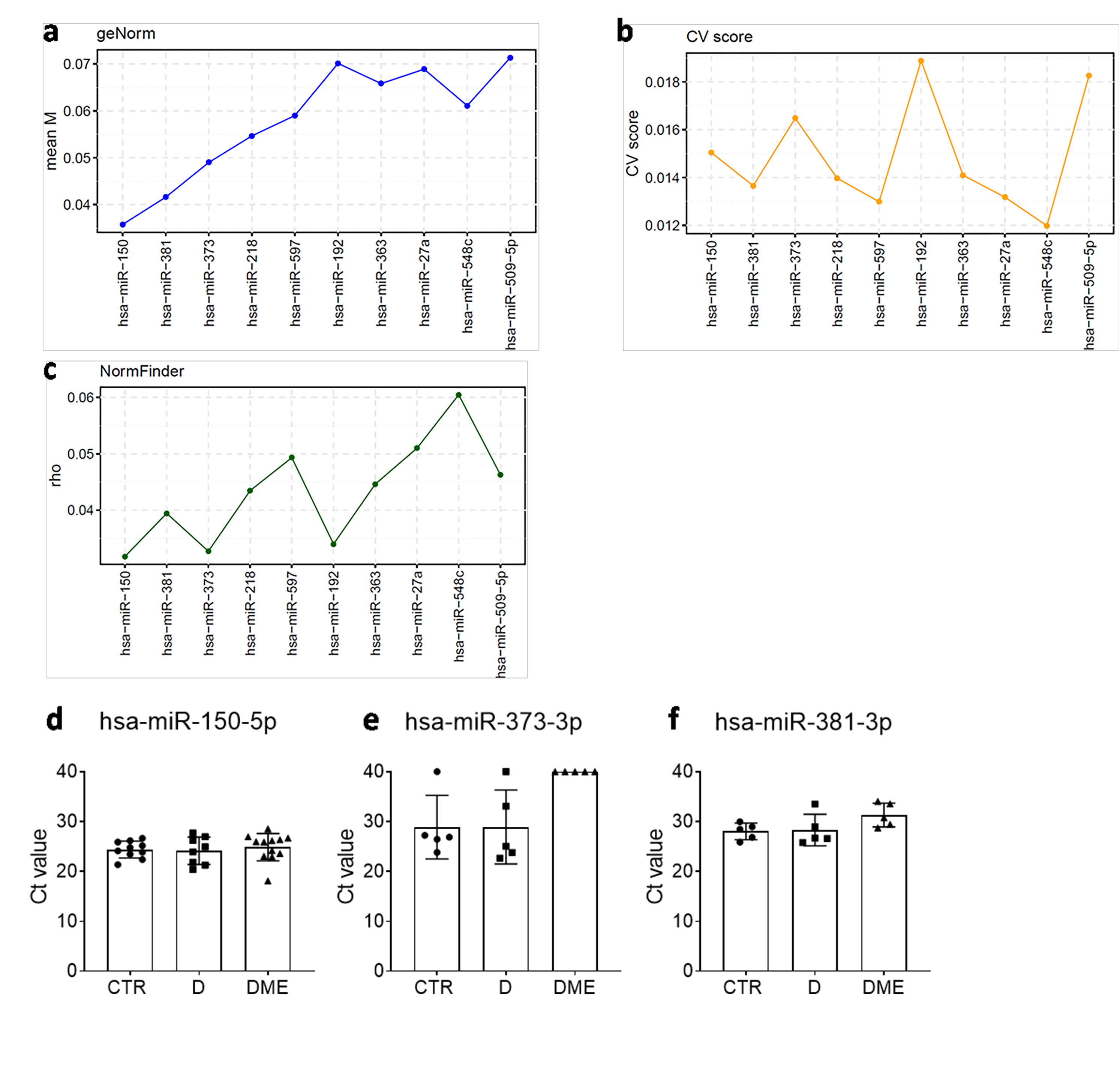

Supplement: Supplementary file 1 [file ijms-21-07328-s001.zip › ijms-942167-supplementary da inviare/Supplementary Figure S1.tif]

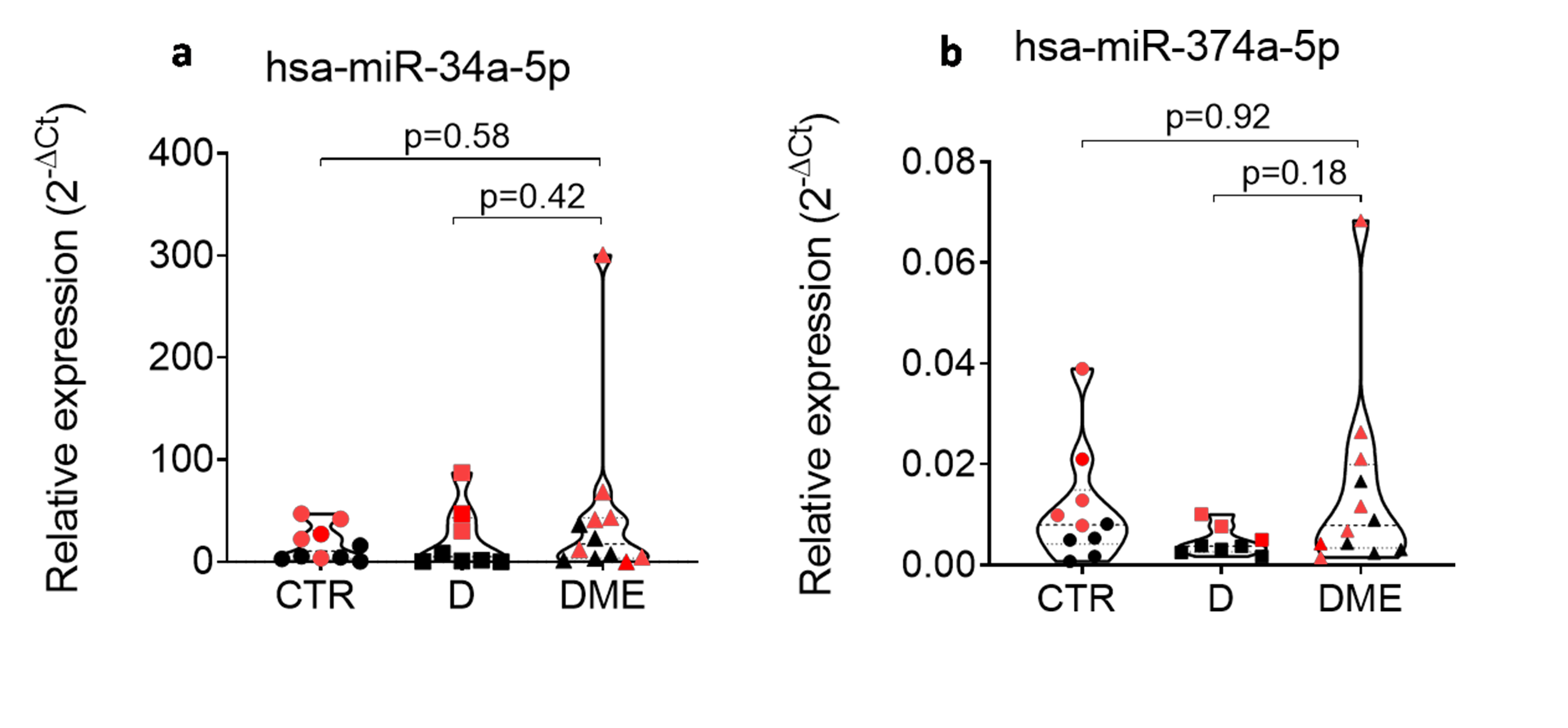

Supplement: Supplementary file 1 [file ijms-21-07328-s001.zip › ijms-942167-supplementary da inviare/Supplementary Figure S2.tif]

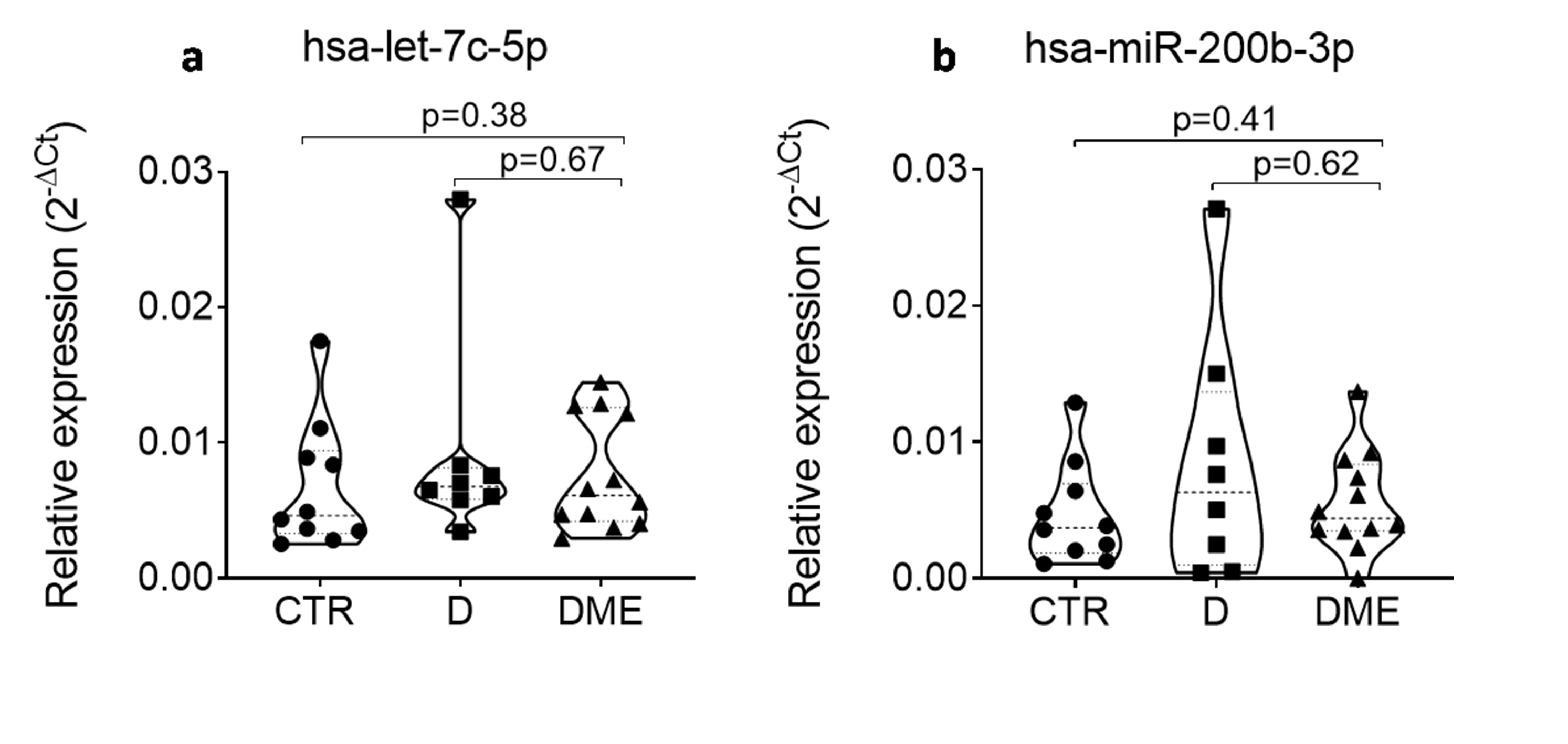

Supplement: Supplementary file 1 [file ijms-21-07328-s001.zip › ijms-942167-supplementary da inviare/Supplementary Figure S3.tif]

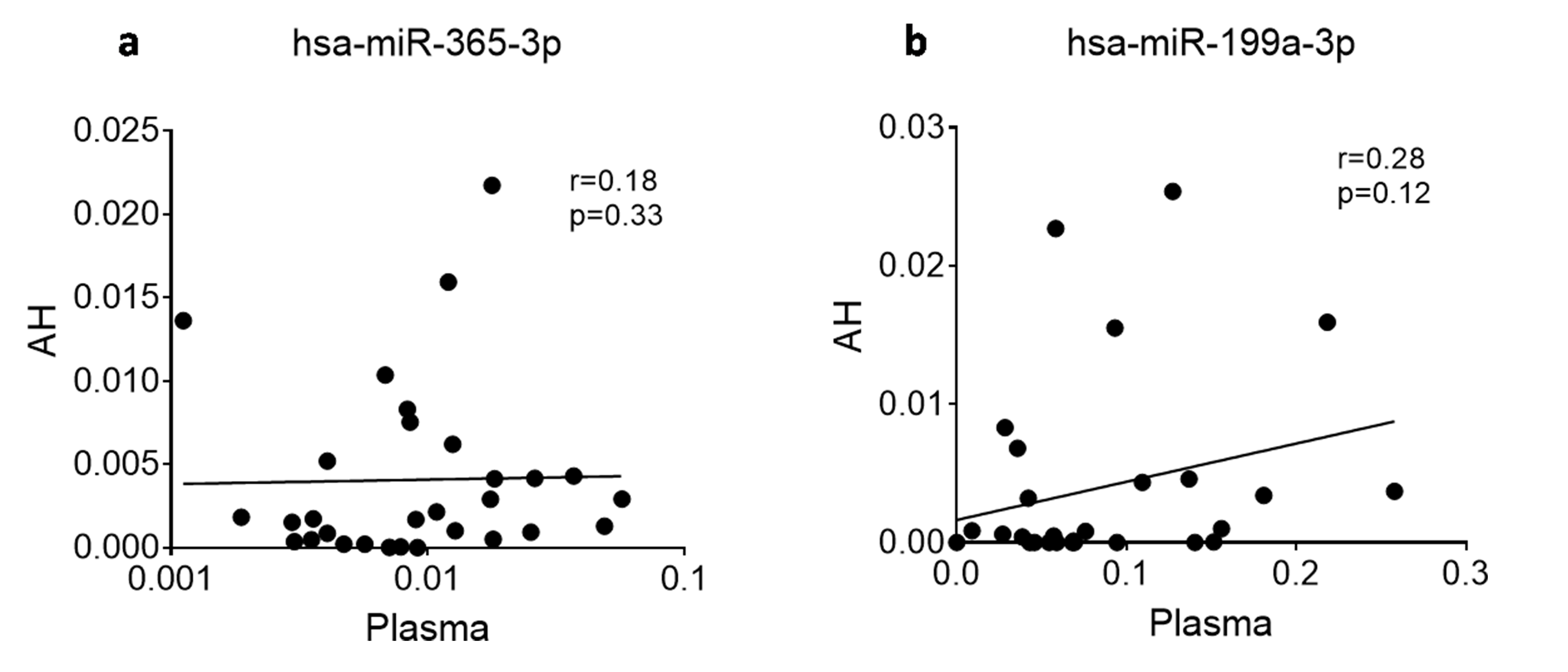

Supplement: Supplementary file 1 [file ijms-21-07328-s001.zip › ijms-942167-supplementary da inviare/Supplementary Figure S4.tif]
